# Deeper sedation during ERCP is associated with fewer retained common bile duct stones: a prospective population-based register study

**DOI:** 10.1186/s12876-026-04742-4

**Published:** 2026-03-21

**Authors:** Eyvind Liljegren, Emma Sverdén, Johanna Österberg, Lars Enochsson, Gabriel Sandblom

**Affiliations:** 1 Department of Clinical Science and Education Södersjukhuset, Karolinska Institute, Sjukhusbacken 10, Stockholm, 118 83 Sweden; 2 Department of Urology, Karolinska University Hospital, Stockholm, Sweden; 3 Department of Surgery, Södersjukhuset, Stockholm, Sweden; 4 Department of Surgery, Mora hospital, Mora, Sweden; 5 Department of Diagnostics and Intervention, Surgery, Umeå University, Umeå, Sweden; 6 Department of Clinical Science, Interventions and Technology, Division of Orthopaedics and Biotechnology, Karolinska Institute, Stockholm, Sweden

**Keywords:** Common bile duct stones, ERCP, Retained CBDS, General Endotracheal Anaesthesia, Monitored Anaesthesia Care, Non anaesthetist administered propofol (NAAP), Balanced propofol sedation (BPS).

## Abstract

**Background:**

Endoscopic retrograde cholangiopancreatography (ERCP) for common bile duct stone (CBDS) removal can be performed under varying levels of sedation. Deeper sedation has been associated with more successful cannulation, but little is known about the effectiveness of ERCP, measured as rate of retained CBDS, depending on the level of sedation.

**Methods:**

In this observational population-based cohort study data on 121 252 ERCP procedures were retrieved from the Swedish Register for Gallstone Surgery and ERCP (Gallriks). Adjusted odds ratios (OR) for retained CBDS 12 months after CBDS removal were estimated. Retained CBDS was defined as reintervention with ERCP finding a CBDS.

**Results:**

General endotracheal anaesthesia (GEA) was associated with 27% lower odds for retained CBDS compared to light endoscopist-directed conscious sedation (EDCS) after adjusting for confounding factors. Adjusted OR for GEA was 0.73 (CI 0.66–0.80), for anaesthetist-directed propofol sedation (monitored anaesthesia care) (ADPS (MAC)) 0.84 (CI 0.76–0.93), and for endoscopist-directed propofol sedation (EDPS) 0.83 (CI 0.72–0.95). Pairwise comparison revealed a 13% (OR 0.87 CI 0.77–0.99) reduction of odds in favour of GEA compared to ADPS (MAC), but no significant difference compared to EDPS.

**Conclusion:**

An association was observed between deeper sedation and lower odds of retained CBDS compared to EDCS, with a marginal benefit of GEA over ADPS (MAC). Safety outcomes were not assessed, and findings are subject to unmeasured confounding why no causal conclusion can be made.

**Supplementary Information:**

The online version contains supplementary material available at 10.1186/s12876-026-04742-4.

## Introduction

Obstruction of the common bile duct (CBD) can lead to complications such as jaundice, cholangitis, or biliary pancreatitis, and is often caused by common bile duct stones (CBDS). The optimal management of CBDS remains a matter of debate, but endoscopic retrograde cholangiopancreatography (ERCP) is widely adopted for stone removal [1]. However, ERCP is a technically challenging procedure and sometimes CBDS are not completely cleared resulting in retained stones and the need for reintervention. 

Cannulation of the Papilla of Vater is a critical first step in the ERCP procedure. There is some evidence that deeper sedation, typically with propofol, is associated with more successful cannulation compared to moderate conscious sedation [2]. However, it remains unclear whether deeper sedation also improves stone removal effectiveness, reducing the long-term risk for retained CBDS.

The challenges of ERCP from a sedation standpoint include the preferred prone position of the patient and the oral approach, and thus proper airway management is essential [3].

The most basic approach to sedation is endoscopist-directed conscious sedation (EDCS), where the patient is sedated using an opioid plus benzodiazepine but remains conscious. Performing ERCP under EDCS can be both time- and resource-efficient for selected patients and procedures, while GEA is considered suitable for more demanding CBDS cases [4].

When propofol is administered without the direct involvement of anaesthesiologists it is sometimes known as non-anaesthetist administered propofol (NAAP), or balanced propofol sedation (BPS). While subtle differences in protocol exist the similar Endoscopist directed propofol sedation (EDPS) aims at moderately sedated patients who have a better experience than EDCS and the goal of sedation is for the patient to remain conscious [5].

More advanced sedation levels, often referred to as monitored anaesthesia care (MAC) or anaesthetist-administered sedation (AAS), are not strictly defined or clearly delineated [5]. However, Anaesthetist-directed propofol sedation (ADPS), similar to MAC and AAS is typically described as deep sedation, as per the continuum of sedation defined by the American Society of Anesthesiologists (ASA) [6, 7].

General endotracheal anaesthesia (GEA) is more clearly defined, as it involves the use of general anaesthesia with endotracheal intubation for airway management, with the optional use of muscle relaxation.

Deep sedation (MAC and GEA) are often preferred for more challenging procedures because of increased patient well-being, fewer interruptions, fewer sedation failures [8], and lower ERCP failure rate compared to EDCS [9]. Arguments could be made that deeper sedation provides the endoscopist with the possibility to achieve a better overview of the bile ducts since patient discomfort does not limit the procedure.

The shift in the role of ERCP from a mainly diagnostic to an increasingly invasive therapeutic procedure, the need for patient comfort, and practical and regulatory factors have resulted in EDCS being largely replaced by deeper sedation or general anaesthesia [5, 10, 11]. However, variations in practice occur and are influenced by factors including healthcare regulations and local tradition. In the United Kingdom, the less resource-intensive EDCS is recommended whenever possible [12], whereas in the USA there has been a shift towards GEA and MAC [10]. In Sweden, most CBDS detected by intraoperative cholangiography at cholecystectomy are extracted intraoperatively using rendezvous ERCP over a guidewire. Thus, many Swedish ERCP procedures to remove stones are registered as being performed under GEA [13, 14].

In an effort to publish consensus guidelines based on recent research [15], the British Society of Gastroenterology concluded that there is a lack of evidence that sedation level affects safety and effectiveness of ERCP. Selection of sedation level remains a balanced clinical decision where conscious sedation should be considered in suitable cases [4, 12]. It is evident that there is a need for further data on the impact of sedation level on ERCP outcome in general, and specifically on the rate of retained CBDS.

We hypothesised that the level of sedation during ERCP is correlated to success of CBDS removal with reduced risk for retained stones.

### Aim

The aim of this study was to evaluate the correlation between sedation level during ERCP and the effectiveness of CBDS removal defined as lack of retained CBDS.

### Method

This observational population-based cohort study was based on data regarding ERCP procedures registered in GallRiks between 1st January, 2007 and 31st December, 2021. GallRiks is a Swedish national register for gallstone surgery and ERCP that has a coverage of more than 90% and is continuously validated. The register has been described in more detail in a previous report [16].

All patients leave informed consent to participate in the register and studies conducted on the data in the register upon scheduling of the ERCP procedure. They are all allowed to withdraw that consent at any time.

The study was approved by the Swedish Ethics Review Authority Dnr 2022-00362-01.

**Fig. 1:**
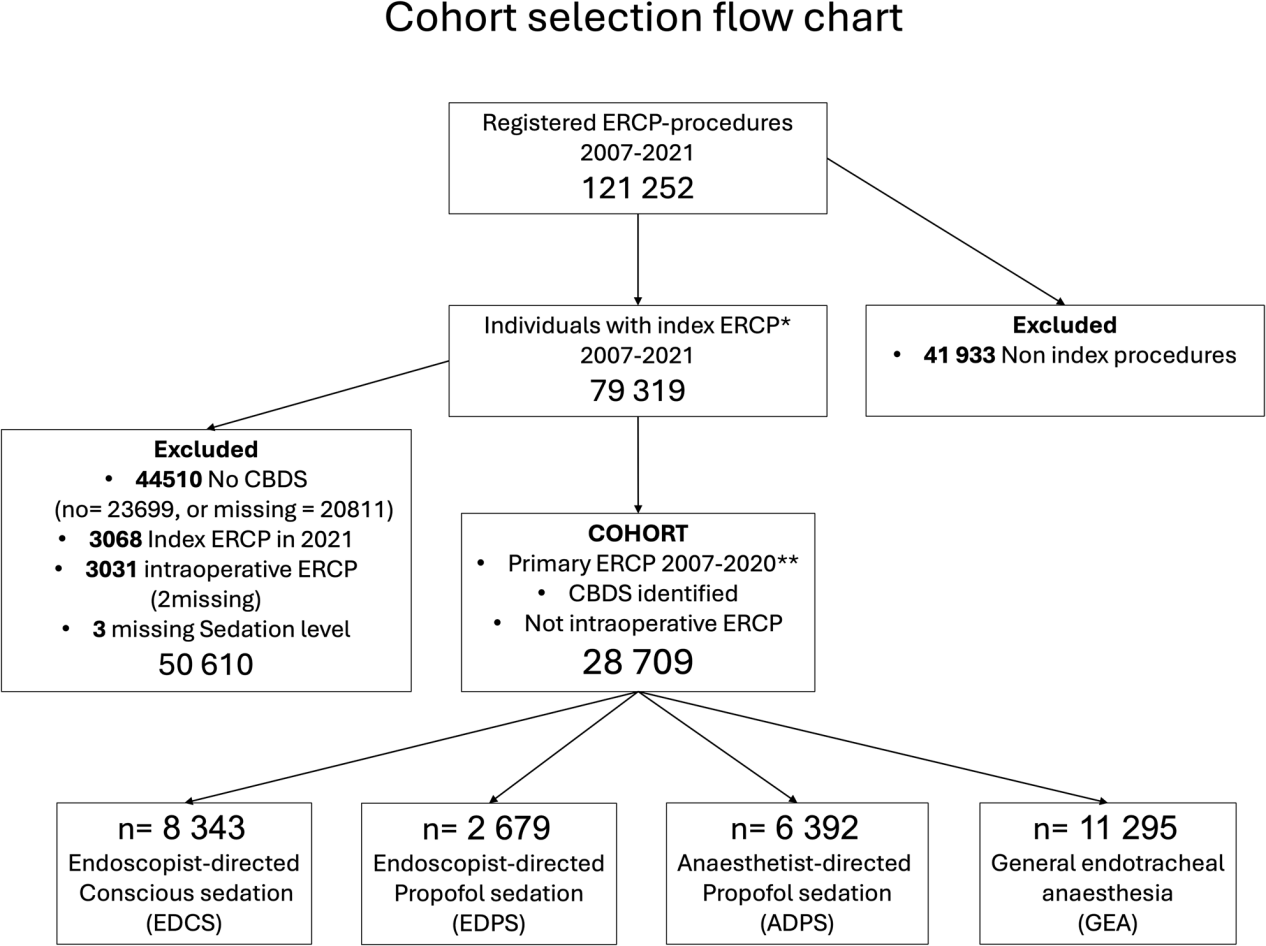
Cohort inclusion flow chart. Detailed in Figure 1 is the cohort inclusion flow chart that describes the selection of the cohort from patients registered in the GallRiks database. * Index ERCP is the first procedure for each individual in the register. ** Patients with index ERCP after January 1st, 2021, were excluded to allow full 12-month follow up

Patients with CBDS identified during the first available ERCP in the register (index-ERCP) performed between 1st January, 2007 and 31st December, 2020 and with complete data on sedation level and intraoperative cholangiogram (IOC) findings were included. Patients with index ERCP after January 1st, 2021, were excluded to allow full 12-month follow-up. Patients who underwent intraoperative ERCP were excluded since GEA was the only option in these cases. Cohort selection is seen in Fig. 1.

### Exposure categories

To maintain transparency and consistency, the sedation levels in this study were categorised in the same way as the four levels of sedation registered in GallRiks:

- *Endoscopist-Directed Conscious Sedation (EDCS)*: defined as minimal to moderate sedation, using a benzodiazepine (commonly midazolam) and an opioid (e.g. fentanyl).
- *Endoscopist-Directed Propofol Sedation (EDPS)*: defined as repeated administration of propofol by a nurse under the direction of the endoscopist, rendering the patient moderately sedated but without need for airway intervention.
- *Anaesthetist-Directed Propofol Sedation (ADPS (MAC))*: defined as deeper sedation and monitoring by an anaesthetist or certified registered nurse anaesthetist [8]. This is comparable to MAC and administered without endotracheal intubation.
- *General Endotracheal Anaesthesia (GEA)*: defined as the deepest level of hypnosis with endotracheal intubation performed by an anaesthetist.

### Outcome measure

A retained CBDS was defined as the presence of a CBDS detected during an ERCP performed within 12 months following the index procedure.

### Follow-up

As the primary outcome was retained CBDS, a 12-month follow-up period was selected to minimise misclassification, taking into account that the majority of retained CBDS cases after cholecystectomy are identified within 18 months, as reported in a previous study based on GallRiks data [17].

Data on index ERCP were collected from 1st January 2007 to 31st December 2020, and outcome data were collected until 31st December 2021.

### Statistical analysis

Statistical analysis and data preparation were performed using open-source statistical software R, R Core Team (2023): A Language and Environment for Statistical Computing. R Foundation for Statistical Computing, Vienna, Austria. <https://www.R-project.org/%3E. in Rstudio, Version 2023.12.0 + 369.

A multivariable logistic regression model was fitted for sedation levels as nominal categorical levels with EDCS as reference.

ORs for retained CBDS at 12 months were calculated in both univariable (unadjusted) and multivariable (fully adjusted) models. Alpha level of 0.05 was considered statistically significant. Point estimates of ORs for each predictor level and confounder are presented as a forest plot with 95% confidence intervals.

### Patient characteristics

Age at index ERCP was categorized as 18–64 years, 65–74 years,75–84 years, and ≥ 85 years to align with previous studies describing risk during endoscopic intervention [18]. Sex was categorised as male or female. ASA class was dichotomised with ASA II or lower considered as mild systemic disease. Gallbladder status was categorised as removed or remaining in situ. Procedure time of index ERCP was categorised as < 30 min (reference), 31–60, and > 60 min. The number of CBDS encountered at index ERCP was categorized as 1 (reference), 2 to 5, 6 to 10 and > 10. The CBDS extraction method was categorized as “balloon and or basket” (reference) or lithotripsy with missing considered a separate level. Shape of the CBD was categorized as normal (reference) or stenosis with missing considered a separate level.

Three additional variables are presented in Table 1 to characterise differences between groups but were not included in the final model. Stent placed during index ERCP was affirmed if a CBD stent was placed at the end of the procedure. Reported complete clearance of CBDS refers to the self-reported success in removal of CBDS at index ERCP and was categorised as “yes”, “incomplete,” or “no”. Deceased during follow-up was categorised as “yes” if the patient was registered in the national population register as dead within 12 months after index ERCP.

**Table 1:**
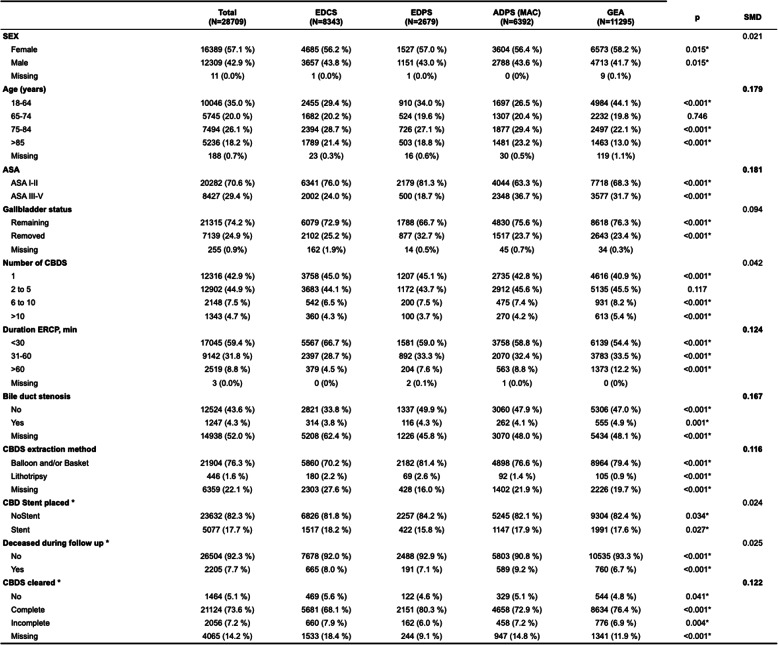
Patient characteristics of cohort with index ERCP showing CBDS grouped by sedation level. *N*=28709

*EDCS* Endoscopist-Directed Conscious Sedation, *EDPS* Endoscopist-Directed Propofol Sedation, *ADPS* (MAC) Anaesthetist-Directed Propofol Sedation (Monitored Anesthesia care), *GEA* General Endotracheal Anaesthesia, *CBDS* Common bile duct stones, *SMD* Standardize Mean Difference (max per variables) calculated from differences in proportions for categorical variables. Higher MSD indicates greater imbalance. compared to group EDCS. *CBD* Common Bile Duct

*P*-values: Chi-square test for nominalvariables with sufficient expected counts, simulated fisher's exact test for variables with low expected counts or binary variables. *P*-values < 0.05 are marked with *

*Not included in adjusted logistic regression

Covariates were added to the final saturated model based on prior clinical knowledge. Missingness was evaluated using Little’s missing completely at random (MCAR) test. Multiple imputation by chained equations (MICE) was implemented where applicable (covariates with low rate of missingness) and modelling of missing as separate level where missingness was significant. Patient characteristics were reported as absolute proportions across predictor strata as well as standardized mean differences (SMD) to emphasize group heterogeneity by reporting the size of the difference. Quantified as SMD from reference variable (EDCS), the covariates’ variation between groups was reported as the maximum SMD across all levels of a covariate. An SMD above 0.1 indicates a meaningful difference between two groups as this threshold is commonly used in observational studies to identify a relevant difference in baseline characteristics [19]. P-values for differences in covariate distribution between the groups, were calculated for completeness while emphasis lies on SMD and descriptive statistics in accordance with modern publication guidelines to avoid overinterpretation of clinically small differences because of large sample size [20, 21].

To assess differences between sedation levels beyond the reference comparison, pairwise comparisons were performed using the *emmeans* package in R. Estimated marginal means were calculated on the log-odds scale while accounting for all covariates in the model, and contrasts were back-transformed and reported as ORs with 95% confidence intervals. P-values were adjusted for multiple comparisons using the Tukey method.

## Results

Of the total 121 252 ERCPs available for analysis, 79 319 unique entries for patients who underwent primary ERCP without any previously registered ERCP during the period of study. Of these, a total of 28 709 had an index ERCP not performed as intraoperative ERCP and with a minimum of one CBDS identified and complete data for level of sedation (Fig. 1).

Among the 20 811 patients with missing data on the number of CBDS at ERCP, 6 307 had suspected or known stone as the reason for index ERCP. Among these, 3,632 were registered as a “normal ERCP”, while 2,432 had unclear findings, and only 205 had a “non-normal ERCP”.

As expected for an observational cohort study, sedation level utilization was to some extent associated with patient-related confounding variables and differences between the groups were observed. All statistical comparisons except for age group 65–74 years and stone count 2–5 showed statistically significant differences. The p-value was far below 0.05, with an absolute majority of covariate levels being unevenly distributed with a p-value < 0.001 (Table 1).

Calculated SMD exceeded the threshold for a relevant difference for covariates age at ERCP (0.179), proportion ASA > II (0.181), duration of ERCP (0.124), bile duct stenosis (0.167) and CBDS extraction method (0.116). Other covariates had SMD indicating a good balance between exposure groups.

Compared with the EDCS group, the GEA group had a higher proportion of patients aged < 65 years (44.0% vs. 29.4%), more procedures lasting > 60 min (12.2% vs. 4.5%), and slightly greater comorbidity with ASA > II (31.7% vs. 24.0%). The proportion of single stones was somewhat lower in the GEA group (40.9% vs. 45.0%), although this difference was small (maximum SMD 0.042).

Patients in the ADPS (MAC) group were older and with higher ASA scores. Compared to GEA they had the gallbladder in situ at a similar rate while single stone cases were slightly more common.

The EDPS group was different with fewer in situ gallbladders (66.7%), lower ASA-score patients (81.3% ASA I-II) and fewer stents placed (15.8%) than all other groups. They also had the highest rate of reported stone clearance, while having an intermediate age distribution.

The EDCS group in turn had the shortest procedure times with only 4.5% having ERCP duration > 60 min.

The cohort average for reported rate of not cleared or incompletely cleared CBDS at index ERCP was 12.3%. There was a difference in this reported success at index ERCP, with the EDCS group reporting the lowest proportion of successful clearance (5 681 of 8 343, 68.1%) and EDPS showing the highest (2 151 of 2679, 80.3%).

The mortality rate during follow-up differed slightly across groups (ADPS (MAC) 9.2%, EDCS 8.0%, GEA 6.7%). Cross-tabulation and p-value calculation using Chi2 test revealed all p-values well below the alpha level of 0.05. The SMD of 0.025 indicates negligible imbalance in mortality between sedation groups (Table 1).

Missingness varied across variables, with CBDS extraction method and bile duct stenosis suffering substantial missingness rates (22.1% and 52% respectively). Missing was treated as a separate category during statistical analysis for these two covariates and it was imputed using MICE for Age at index ERCP and for Gallbladder status. Missing also varied significantly between sedation levels for CBDS cleared, that was not included in the final model. 

Among the 28 709 patients analysed, a total of 3780 (13.2%) had retained stones, defined as a repeat ERCP with an identified CBDS within twelve months. In the EDCS group, the proportion with retained CBDS was 1 200 of 8 343 (14.4%), while EDPS had 330 of 2 679 (12.3%), ADPS (MAC) had 842 of 6 392 (13.2%) and GEA had 1 408 of 11 295 cases (12.5%).

Over the studied period, the practice of sedation changed, with EDCS and even more so EDPS being increasingly replaced by GEA in recent years. This shift coincides with a slight downward trend in the overall retained stone rate and by logistic regression, the odds of retained CBDS decreased annually during the study period. (OR per year = 0.99, 95% CI 0.98–1.00; *p* = 0.01) (Figure S1).

In univariable logistic regression, the likelihood of retained CBDS was lower when reported bile duct clearance at ERCP was complete (OR 0.08, 95% CI 0.07–0.09; *p* < 0.001). Interestingly, incomplete clearance showed a higher odds of retained stones than those reported as having no clearance. Detailed rates of retained stones for each reported clearance category are provided in Supplementary Table S1.

Missing has been modelled as a separate level. Unadjusted univariable logistic regression results for all other covariates of Table 1 are available in Table S2.

Table S1 describes the distribution across levels of reported CBDS clearance at index ERCP cross tabulated with the rate of and OR for retained CBDS at 12 months. CI=95% confidence interval

Unadjusted logistic regression with EDCS as reference demonstrated lower odds of having a retained CBDS for all levels of deeper sedation: EDPS (OR 0.84 (95% CI 0.73–0.95)); ADPS (MAC) (OR 0.90 (95%CI 0.82–0.99)); and GEA (OR 0.85 (95%CI 0.78–0.92)) (Table 2).

**Table 2:** Unadjusted and adjusted OR of retained CBDS at 12 months by sedation level

Sedation Level	Unadjusted OR	95% CI	*p*		Adjusted OR*	95% CI	*p*	
EDCS								
EDPS	0.84	(0.73, 0.95)	0.007	*	0.83	(0.72, 0.95)	0.006	*
ADPS (MAC)	0.90	(0.82, 0.99)	0.035	*	0.84	(0.76, 0.93)	< 0.001	*
GEA	0.85	(0.78, 0.92)	< 0.001	*	0.73	(0.66, 0.8)	< 0.001	*

SEX, AGE at index ERCP in years (18–64), ASA classification (I-II), Gallbladder (remaining), Number of CBDS at index [1], ERCP duration in 30 min increments (< 30 min), Bile duct stenosis (No), CBDS extraction method (Balloon and/or basket)

*EDCS* Endoscopist-Directed Conscious Sedation (reference), *EDPS* Endoscopist-Directed Propofol Sedation, *ADPS* (MAC) Anaesthetist-Directed Propofol Sedation, *GEA* General Endotracheal Anaesthesia, *OR* Odds ratio, *CBDS* Common bile duct stones

**Fig. 2:**
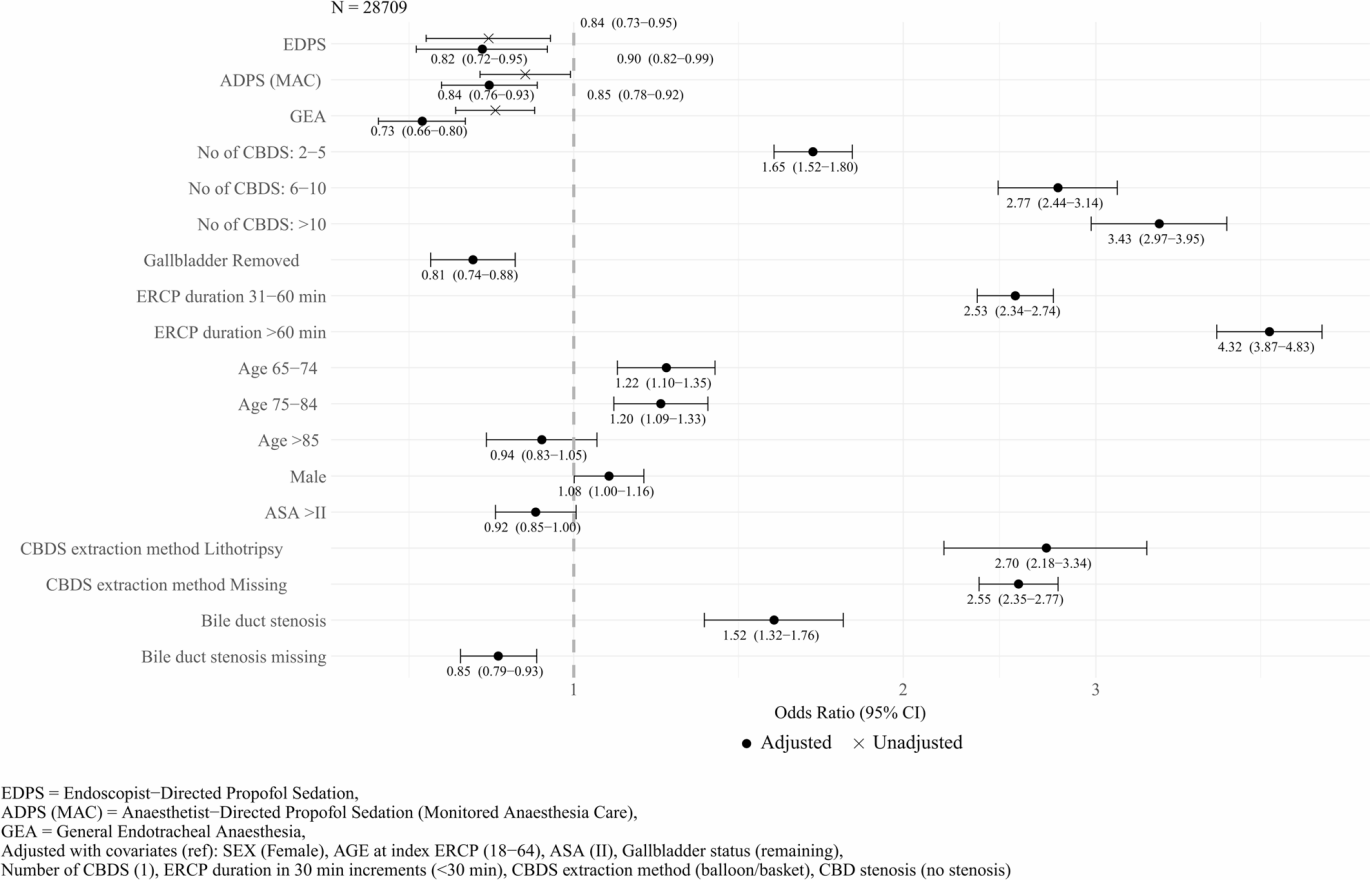
Forest plot of ORs for sedation levels and covariates in the fully adjusted model and for sedation levels in the unadjusted model

*Adjusted OR in the fully adjusted model with covariates (ref):

In the adjusted multivariable logistic regression model, the OR for retained CBDS at twelve months in the GEA group was 0.73 (95% CI 0.66–0.80) and in the ADPS (MAC) group 0.84 (95% CI 0.76–0.93), compared to EDCS. EDPS had an estimated OR of 0.83 (95%CI 0.72–0.95) (Table 2). Unadjusted ORs as well as the predictors impact on the OR in the adjusted model is shown in Fig. 2.

Furthermore, pairwise comparisons of sedation levels using estimated marginal means from the fully adjusted logistic regression model demonstrated a significant difference in the odds of the outcome for GEA compared with ADPS (MAC), with Tukey-adjusted p-values and marginal means averaged over all other covariates (Table 3). No significant difference between other levels of deeper sedation was observed.

**Table 3:** Pairwise comparisons of sedation levels calculated from estimated marginal means in the fully adjusted* model

Compared sedation levels	OR	95% CI	*p*	
ADPS (MAC) vs. EDPS	1.01	0.84–1.22	0.9970347	
GEA vs. EDPS	0.88	0.74–1.05	0.2603569	
GEA vs. ADPS (MAC)	0.87	0.76–0.99	0.0225097	*

SEX, AGE at index ERCP in years (18-64), ASA classification (I-II), Gallbladder (remaining), Number of CBDS at index [1], ERCP duration in 30 min increments (<30 min), Bile duct stenosis (No), CBDS extraction method (Balloon and/or basket)

*EDCS* Endoscopist-Directed Conscious Sedation (reference), *EDPS* Endoscopist-Directed Propofol Sedation, *ADPS* (MAC) Anaesthetist-Directed Propofol Sedation,*GEA* General Endotracheal Anaesthesia,*OR* Odds ratio, *CBDS* Common bile duct stones

*Adjusted OR in the fully adjusted model with covariates (ref):

Table 3 details the pairwise comparison between sedation levels and the OR of retained CBDS stones calculated via marginal means from the fully saturated model.

## Discussion

In this observational population-based cohort study using prospectively collected register data, 13.2% of patients had retained CBDS within 12 months of attempted clearance by ERCP, consistent with a recent meta-analysis reporting an overall rate of 13% of retained CBDS after ERCP [22].

This study observed the use of GEA to be associated with a 27% reduction in adjusted odds of retained CBDS compared to EDCS, with observed reductions of (16%) for ADPS (MAC) and EDPS (18%) respectively. The overlapping confidence intervals and non-significant pairwise comparisons between ADPS (MAC) and EDPS groups indicate no difference in association between propofol sedation and retained CBDS regardless of who supervises it.

In pairwise comparison, GEA was statistically significantly associated with 13% lower odds than ADPS (MAC). The confidence interval, however, approached unity with upper bound of 0.99 indicating some uncertainty regarding this observed difference in association with retained CBDS between the two sedation levels.

Although no previous studies have specifically examined rates of retained stones in relation to sedation level, other publications have reported similar rates of technical success and procedure interruptions when comparing GEA with ADPS (MAC) [4, 10, 23], which somewhat contrasts the observed difference in favour of GEA in this present study.

The observed significant association between all deeper sedation levels and lower odds of retained CBDS compared to EDCS in this study are more in line with previous findings of improved success with deeper sedation when comparing propofol sedation to basic sedation comparable to EDCS [2, 10].

A trend towards increased use of GEA and ADPS (MAC) over EDCS, as seen in U.S. data, was also evident in our cohort, albeit with a time lag [10]. EDPS, (in other publications referred to as NAAP or BPS), was the least frequently used sedation strategy in our cohort (*n* = 2 679). The available literature on EDPS is heterogeneous, and data on retained stone rates are lacking. However, studies comparing propofol-based sedation with non-propofol sedation administered by non-anaesthesiologists have generally favoured propofol with respect to patient experience, while safety outcomes appear comparable [24].

While this study did not address complications separately, ERCP under GEA has previously been associated with fewer sedation-related adverse events in high-risk patients, whereas EDCS has been associated with more frequently interrupted procedures. However, data on sedation-related adverse events stratified by sedation level during ERCP is somewhat limited outside of the high-risk populations [3, 6, 8, 25].

Patients without a gallbladder at index ERCP had lower unadjusted odds of retained stones (OR 0.88, 95% CI 0.77–1.00) compared with those with a gallbladder in situ, suggesting that a proportion of stones classified as retained may represent early recurrence originating from the gallbladder rather than a retained CBDS that was overlooked at index ERCP. However, detailed gallbladder stone status was unavailable in the register, introducing residual confounding and limiting the ability to distinguish true retained stones from recurrent gallbladder-derived stones.

Mortality during follow-up differed slightly between groups, with the highest rate observed in the ADPS (MAC) group. As ERCP-related complications were not included in the model, any interpretation of the cause of this increase remains speculative. The ADPS (MAC) group was older and had higher ASA scores, which could theoretically contribute to higher mortality from natural causes. Censoring bias should also be considered, as older or frailer patients may be managed with less invasive options—such as percutaneous drainage or expectant management—for retained CBDS, and therefore might not undergo repeat ERCP.

Longer procedure time correlated with increased odds of retained stones, possibly reflecting higher complexity. Deeper sedation may theoretically facilitate more thorough clearance, potentially explaining longer duration under GEA. Conversely, more proficient operators at high-volume centres could bias this association if their routine was to favour GEA over other sedation methods. This remains unknown as our data did not include care provider identification.

The prevalence of stenosis of the CBD is not widely reported in the literature but in one cohort stenosis was seen in 2–5% [26], roughly corresponding to the available data in the present study with rates at 4.3%. This lends some credibility to the idea to treat missing as a negative, although we opted for separate category encoding of missing in the analysis.

For CBDS removal by lithotripsy, the rate in the present study is even more sparse with only 1.6% confirmed and 22% missing. This is a much lower rate of lithotripsy than in available literature with reports of up to 10–20% of CBDS cases, although mainly for complicated cases [27], suggesting either underreporting in the register or true difference of practice.

Including stenosis and lithotripsy in the adjustment attenuated the reduction in odds for all sedation levels slightly when compared to adjusted model without them, but did not alter the overall conclusion or direction of association on sensitivity analysis (tabulated robustness data available on request).

Stent placement at end of ERCP, although a marker of retained stones because many ERCPs are scheduled as a second session to finish removal of stones and stent, was excluded from the adjusted model due to collinearity but is presented descriptively. Similarly, endoscopist-reported clearance was excluded from modelling but included in Table 1 and separate covariate univariable regression.

When a patient was reported as “completely cleared” of CBDS at index ERCP it was associated with 92% lower odds of suffering a retained stone. Even though dramatically lower, the rate of retained stones at one year was still 6.2% among these patients. This indicates that some cases of missed stones or misclassification may have occurred where clearance was thought to be complete at index ERCP but stones were later found at reintervention.

Furthermore, the retained stone rate was somewhat higher among the patients with missing data for duct clearance status than in the “completely cleared”. This could indicate informative missing with uncleared or incompletely cleared stones being overrepresented among those with missing.

Strengths of this study include the large sample size, minimal loss to follow-up, and near-complete national coverage. Leveraging repeat ERCP for CBDS as a definition for retained CBDS is a robust outcome measure with clear clinical implication, lending further relevance to the findings. A 12-month follow-up window allows balance between detection of clinically relevant retained stones with minimal misclassification of de novo stones.

### Limitations

Selection bias and residual unmeasured confounding are inherent limitations of this observational study.

Important markers of procedural complexity such as stone size, CBD anatomy, cholangitis, coagulopathy, and anticoagulant or antiplatelet use were not available in the registry and could therefore not be included in the model.

If more complex cases are preferentially managed under deeper sedation while simultaneously carrying a higher intrinsic risk of retained stones, unmeasured residual confounding caused by case complexity may bias the observed association with retained stones toward the null and attenuate the apparent benefit of deeper sedation.

Unmeasured confounding in this study may therefore bias the estimated ORs towards underestimating the association between GEA and reduced stone odds. Some support for this reasoning is the fact that the case complexity markers that are included (stone count, bile duct stenosis, CBDS extraction method) in the model drive the associated OR of retained stone in favour of GEA compared to unadjusted estimates.

Residual confounding limits interpretation to associations rather than causal inference and may also affect generalisability.

Several covariates of potential interest were available in the GallRiks but were not included in this adjusted logistic regression. Cholangiographic findings, for example, are only recorded during cholecystectomy and not during ERCP, and could therefore not be used. Difficult cannulation was only documented from 2016 onwards, making it ineligible for inclusion without losing a substantial amount of data. Future analyses covering this period will be able to leverage the full set of modern variables. Stone location in the biliary ducts could be analysed from 2016 onwards, so it was not available for the entire study period. Moreover, CBDS location was considered unlikely to substantially bias sedation selection, and subgroup analysis was therefore omitted.

Among the 6 307 patients with suspected CBDS as the indication for ERCP but missing CBDS status, stones may have been present but were not included in the analysis of retained CBDS, as no stones were documented at the index ERCP. Any resulting bias cannot be quantified; however, only 205 of these patients had abnormal ERCP findings and 2 432 had unclear findings, suggesting that any impact on the estimated outcomes is likely limited.

The increased use of GEA for sedation during ERCP, as shown in Figure S1, may introduce temporal confounding if general improvements in patient care have also enhanced the effectiveness of stone management. The very small reduction in odds of retained CBDS described by regression on treatment year as explanatory variable indicates that this effect is likely small and that statistical significance in a very large dataset like this does not necessarily reflect a clinically meaningful effect. Future studies that account for different time intervals could help clarify the potential temporal confounding in this study.

## Conclusion

Deeper sedation (GEA, ADPS (MAC) and EDPS) was associated with lower odds of retained CBDS after ERCP compared with conscious sedation.

This large register-based cohort study does demonstrate an association between lower odds of retained CBDS at 12 months for GEA compared to ADPS (MAC) as well as for all levels of deep sedation versus EDCS.

However, unmeasured confounding and potential selection bias preclude causal inference.

Sedation-related adverse events were not analysed, and the choice of sedation strategy during ERCP for CBDS removal therefore remains a balanced clinical decision until further randomised trials clarify any causal relationship between sedation level and success of stone clearance at ERCP.

## Supplementary Information

Supplementary Material 1. Figure S1. Sedation levels and their proportional utilization by year as stacked bars and retained stone rates per year per sedation level as lines. Supplementary Material 2. Table S1 – Rate of retained CBDS by reported clearance of CBDS at index ERCP. Supplementary Material 3. Table S2 - Overview of all covariates and corresponding univariable logistic regression with OR of retained CBDS within 12 months.

## Data Availability

Pseudonymised data and R-script are made available on request from the corresponding author.

## Abbreviations

ADPS (MAC): Anaesthetist-directed propofol sedation (monitored anaesthesia care)
ASA: American Society of Anesthesiologists
BPS: Balanced propofol sedation
CBD: Common bile duct
CBDS: Common bile duct stones
CI: Confidence interval
EDCS: Endoscopist-directed conscious sedation
EDPS: Endoscopist-directed propofol sedation
ERCP: Endoscopic retrograde cholangiopancreatography
GallRiks: Swedish Register for Gallstone Surgery and ERCP
GEA: General endotracheal anaesthesia
IOC: Intraoperative cholangiography
MCAR: Missing completely at random
MICE: Multiple imputation by chained equations
NAAP: Non-anaesthetist administered propofol
OR: Odds ratio
SMD: Standardized mean difference
