# Supplementary material for: Deeper sedation during ERCP is associated with fewer retained common bile duct stones: a prospective population-based register study
Source: BMC Gastroenterol. 2026 Mar 21;26:212. doi: 10.1186/s12876-026-04742-4 (PMC13063613; doi:10.1186/s12876-026-04742-4)

Figure S1


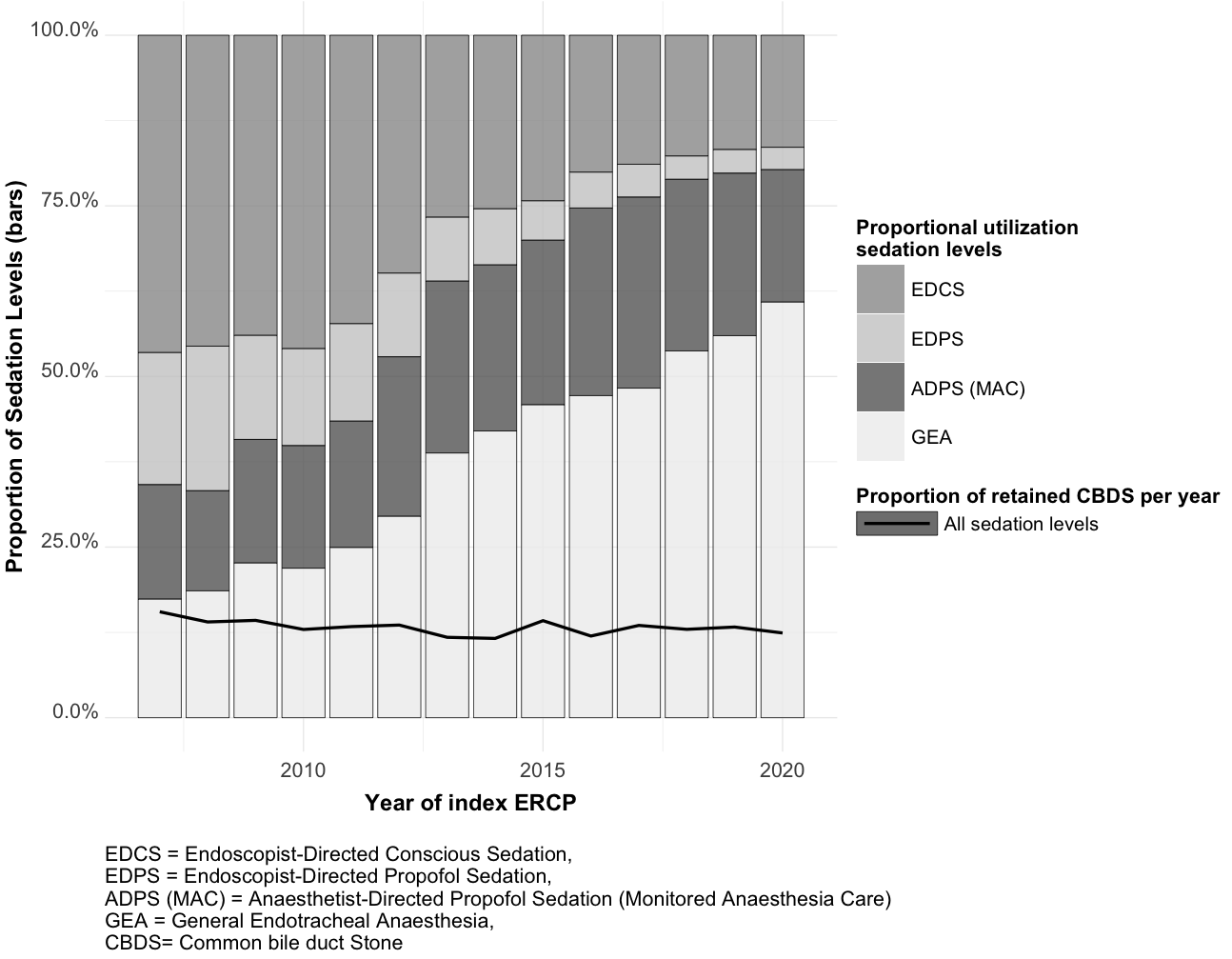
Sedation levels and their proportional utilization by year as stacked bars and retained stone rates per year per sedation level as lines

Table S1 – Rate of retained CBDS by reported clearance of CBDS at index ERCP

|  | | | | | | |
| --- | --- | --- | --- | --- | --- | --- |
| **Reported clearance** | **n** | **Retained CBDS, n** | **Retained CBDS (%)** | **OR** | **CI** | **p** |
| No | 1464 | 667 | 45.6 | 1.000 | (reference) |  |
| Complete | 21124 | 1315 | 6.2 | 0.079 | (0.07, 0.09) | <0.001 |
| Incomplete | 2056 | 1197 | 58.2 | 1.665 | (1.45, 1.91) | <0.001 |
| Missing | 4065 | 601 | 14.8 | 0.207 | (0.18, 0.24) | <0.001 |

Table S2 - Overview of all covariates and corresponding univariable logistic regression with OR of retained CBDS within 12 months.


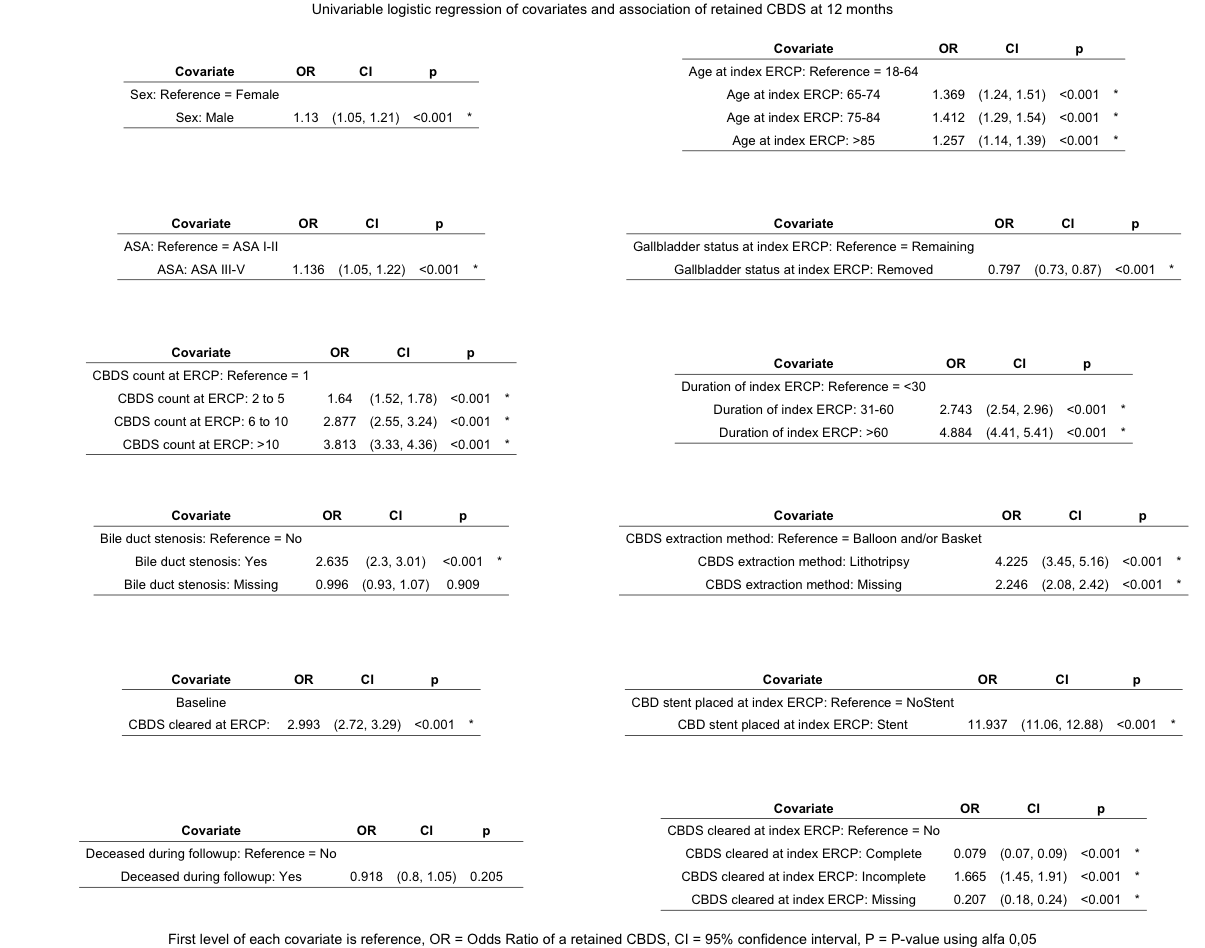

Supplement: Supplementary file 1 — Supplementary Material 1. Figure S1. Sedation levels and their proportional utilization by year as stacked bars and retained stone rates per year per sedation level as lines. Supplementary Material 2. Table S1 – Rate of retained CBDS by reported clearance of CBDS at index ERCP. Supplementary Material 3. Table S2 - Overview of all covariates and corresponding univariable logistic regression with OR of retained CBDS within 12 months. [file 12876_2026_4742_MOESM1_ESM.docx]
